# Towards antiviral polymer composites to combat COVID‐19 transmission

**DOI:** 10.1002/nano.202100078

**Published:** 2021-05-04

**Authors:** Adrian P. Mouritz, Joel Galos, Denver P. Linklater, Raj B. Ladani, Everson Kandare, Russell J. Crawford, Elena P. Ivanova

**Affiliations:** ^1^ School of Engineering RMIT University GPO Box 2476 Melbourne Victoria 3001 Australia; ^2^ School of Science RMIT University GPO Box 2476 Melbourne Victoria 3001 Australia

**Keywords:** antiviral surfaces, multifunctional composites, nanomaterials

## Abstract

Polymer matrix composite materials have the capacity to aid the indirect transmission of viral diseases. Published research shows that respiratory viruses, including severe acute respiratory syndrome coronavirus 2 (SARS‐CoV‐2 or COVID‐19), can attach to polymer substrata as a result of being contacted by airborne droplets resulting from infected people sneezing or coughing in close proximity. Polymer matrix composites are used to produce a wide range of products that are “high‐touch” surfaces, such as sporting goods, laptop computers and household fittings, and these surfaces can be readily contaminated by pathogens. This article reviews published research on the retention of SARS‐CoV‐2 and other virus types on plastics. The factors controlling the viral retention time on plastic surfaces are examined and the implications for viral retention on polymer composite materials are discussed. Potential strategies that can be used to impart antiviral properties to polymer composite surfaces are evaluated. These strategies include modification of the surface composition with biocidal agents (e.g., antiviral polymers and nanoparticles) and surface nanotexturing. The potential application of these surface modification strategies in the creation of antiviral polymer composite surfaces is discussed, which opens up an exciting new field of research for composite materials.

## Graphical abstract

Polymer matrix composites are used to produce a wide range of products that are “high‐touch” surfaces, such as sporting goods, laptop computers and household fittings, and these surfaces can be readily contaminated by pathogens. Factors controlling the viral retention time on plastic surfaces including SARS‐CoV‐2 (COVID‐19) are examined and the implications for viral retention on polymer composite materials are discussed. Potential strategies that can be used to impart antiviral properties to polymer composite surfaces are evaluated.

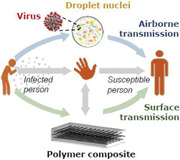

## 1 INTRODUCTION

**FIGURE 1:**
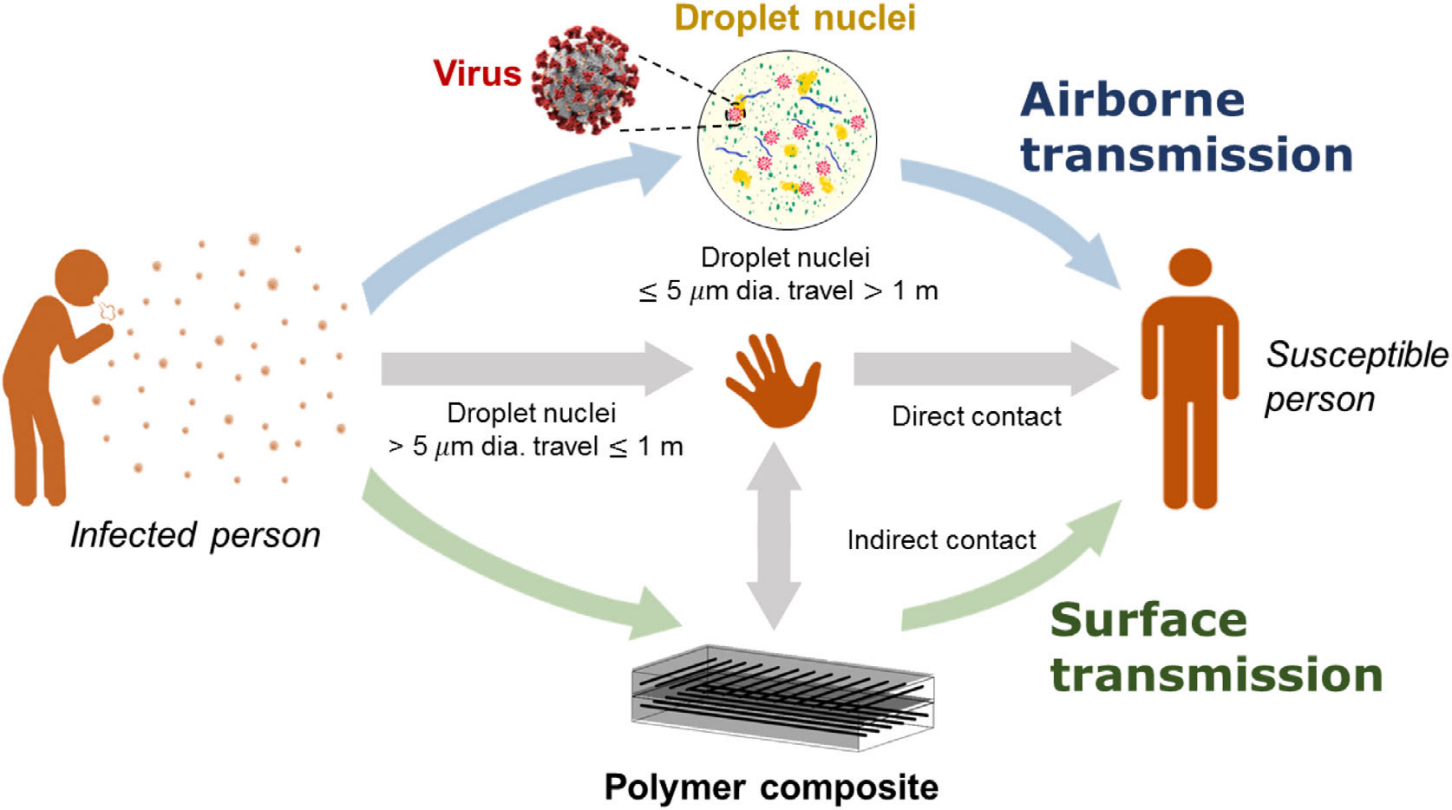
Transmission routes: airborne, droplets, direct contact, and indirect contact from surfaces

SARS‐CoV‐2 or COVID‐19 is a highly contagious pathogen that is transmitted between humans mainly via respiratory droplets (typically <1‐2000 μm in diameter).^[^ ^1^, ^2^, ^3^, ^4^ ^]^ Indirect transmission of SARS‐CoV‐2 also occurs via contamination of abiotic surfaces; respiratory droplets from an infected human can inadvertently be deposited onto surfaces via sneezing or coughing, resulting in new infections when the contaminated surfaces are then touched by others.^[^ ^5^, ^6^, ^7^ ^]^ Human fingers that touch a virus‐laden surface can spread the virus to up to seven other clean surfaces.^[^ ^8^ ^]^ Infection of the next host occurs when the virus is again transferred by touching their mouth, nose or eyes. The common transmission pathways, including indirect transmission via surfaces (so‐called “hidden transmission”^[^ ^9^ ^]^), are schematically represented in Figure 1. Indirect transmission of SARS‐CoV, MERS‐CoV, influenza viruses and other respiratory pathogens via abiotic surfaces is exacerbated by the fact that viral particles can survive for extended periods, often for many hours or days under suitable conditions.^[^ ^6^, ^7^, ^9^, ^10^, ^11^, ^12^, ^13^, ^14^ ^]^ However, instances of indirect viral transmission via abiotic surfaces are believed to be small compared to the spread of infections by direct transmission.^[^ ^9^ ^]^ The contribution of indirect transmission of SARS‐CoV‐2 to its rapid spread, leading to the global pandemic, is unknown. Despite this uncertainty, major global efforts are being made to decontaminate surfaces by deep‐cleaning and disinfection of “high‐touch” surfaces in hospitals, workplaces, public transport and community spaces (among others) as a means of reducing the spread of SARS‐CoV‐2.

Fiber‐reinforced polymer composite materials are used extensively in the production of objects with “high‐touch” surfaces, such as mobile phone cases, sporting goods, bathroom and kitchen fittings, and the interior surfaces of rail carriages, boats and passenger aircraft. Reducing the risk of indirect transmission of viruses via the surface of polymer composite materials is, therefore, a crucial public health issue. To the best of our knowledge, there is no published research into determining the ability for viruses to survive on polymer composite materials, which is an important factor in establishing the risk of indirect transmission from an infected person to an infection‐susceptible person (Figure 1). Similarly, there is no published research into methods by which composite surfaces can be modified in order to reduce the potential risk for indirect virus transmission. Nevertheless, several reports have shown that viruses (usually residing within droplets) can be retained on plastic surfaces. A number of surface treatment methods have been proposed that have the potential to reduce the retention of viruses on plastics; these techniques could be applied to polymer composite materials in order to reduce the risk the chances of indirect transmission of viruses; however, this is yet to be tested.

This review examines published research into the retention and survival of SARS‐CoV‐2 and related viruses on polymer surfaces, the factors that influence the retention of viruses, including SARS‐CoV‐2, on plastics, and discusses the applicability of this research to polymer composite materials. We critically appraise the various surface treatment methods that have the potential to impart antiviral properties to polymer composite materials. Such techniques include the addition of polymers and metals (such as copper and silver) with intrinsically antiviral properties or biocidal doping agents to the composite matrix, embedding of nanoparticles within the surface layer, and direct surface modification by the process of nanotexturing. Furthermore, we identify the critical areas and perspectives for further research into controlling and reducing the potential risks presented by polymer composite materials in the transmission of viral diseases such as COVID‐19.

## 2 VIRAL CONTAMINATION OF POLYMER AND POLYMER COMPOSITE SURFACES

**FIGURE 2:**
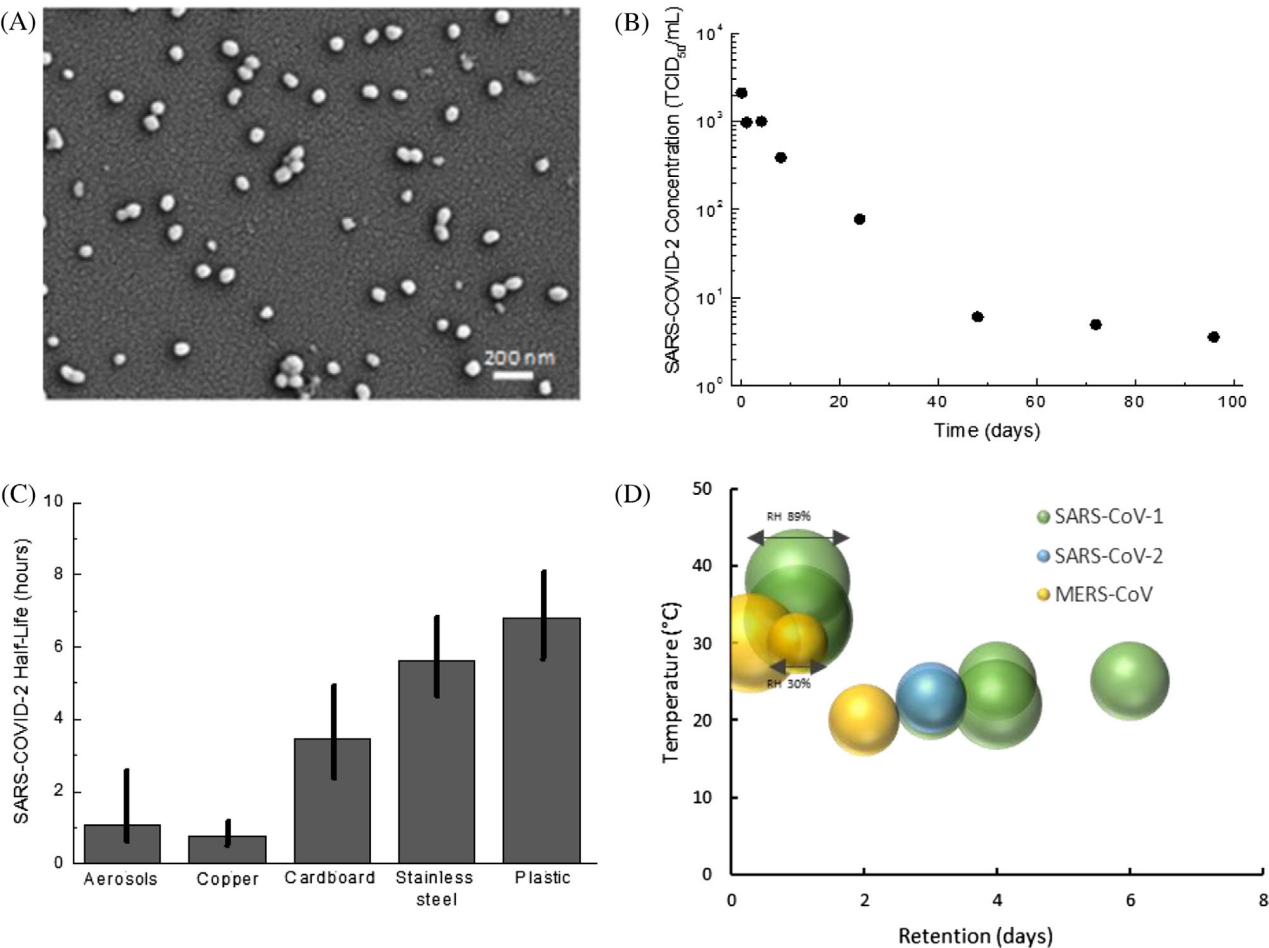
A, Influenza A viral strain PR8H on a silicon surface. B, The concentration of SARS‐CoV‐2 on plastic (polypropylene) as a function of time. Data from.^[^ ^16^ ^]^ C, Comparison of the half‐life of SARS‐CoV‐2 on different surfaces. The vertical line indicates the variability in the measured half‐life, with the maximum and minimum being the 2.5‐97.5% quartile range. Data from.^[^ ^16^ ^]^ D, The retention in days of SARS‐CoV‐1, SARS‐CoV‐2 and MERS‐CoV on plastic surfaces as a function of temperature and relative humidity (RH). Data retrieved from^[^ ^18^, ^23^, ^28^, ^29^ ^]^

The survivability of viruses on surfaces have been investigated for a variety of materials (mostly those found in hospital and other health care facilities), including metals (e.g., aluminum,^[^ ^15^ ^]^ copper,^[^ ^7^, ^16^, ^17^, ^18^ ^]^ steel^[^ ^7^, ^16^, ^17^, ^19^, ^20^ ^]^), glass,^[^ ^18^, ^21^ ^]^ cardboard/paper,^[^ ^7^, ^16^, ^21^ ^]^ cloth materials (e.g., surgical masks, gowns),^[^ ^21^, ^22^, ^23^, ^24^ ^]^ latex/rubber,^[^ ^15^, ^19^, ^25^ ^]^ polystyrene^[^ ^26^ ^]^ and other plastics.^[^ ^7^, ^16^, ^17^, ^18^, ^19^, ^21^, ^25^, ^26^, ^27^ ^]^ An example of an influenza virus on the surface of silicon is shown in Figure 2. The survival of viruses on materials commonly handled between people, such as banknotes, has also been studied.^[^ ^28^ ^]^ Most of the materials examined in these studies are used in hospitals, personal protective equipment or medical wear, wherein the risk of indirect transmission is high. Indirect virus transmission from a carrier to other people via a contaminated surface depends on several factors, most notably the ability for the virus to be retained on the surface of the material. Respiratory viruses are ejected from infected persons via droplets of saliva and nasal discharge that can adhere to surfaces.^[^ ^29^ ^]^ The half‐life of viruses, which is dependent on the virus type and concentration, surface conditions, mode of deposition, temperature and relative humidity, has also been studied.^[^ ^13^, ^14^, ^30^, ^31^ ^]^

The retention of SARS‐CoV‐2 and other viral species is sensitive to the physical condition of the surface. In general, the virus half‐life is shorter on porous materials (e.g., tissue, cloth) than on non‐porous materials (e.g., metals and plastics).^[^ ^7^, ^16^, ^28^, ^30^, ^32^ ^]^ For example, Chin et al.^[^ ^28^ ^]^ recently measured large differences in the retention of SARS‐CoV‐2 between selected porous and non‐porous materials under ambient conditions (22°C, 65% relative humidity (RH)). It was found that SARS‐CoV‐2 persisted on porous materials such as tissue paper for up to ∼3 hours, yet had the ability to survive on non‐porous surfaces, such as plastic, for approximately 7 days. In another study, it was found that SARS‐CoV‐1 had the ability to survive on porous paper for less than 1 day,^[^ ^23^ ^]^ on porous cotton for between 5 minutes and 1 day,^[^ ^23^ ^]^ on non‐porous glass for up to 5 days,^[^ ^21^ ^]^ and on plastic for 4‐5 days.^[^ ^7^, ^16^, ^21^ ^]^

Van Doremalen et al.^[^ ^16^ ^]^ and Suman et al.^[^ ^7^ ^]^ measured the survival period of SARS‐CoV‐2 on copper, stainless steel, cardboard and plastic. The change in the concentration of SARS‐CoV‐2 on a plastic surface over time is shown in Figure 2B.^[^ ^16^ ^]^ Under ambient conditions, the concentration of an active virus on a plastic surface decreases at a non‐linear rate as a function of time before being completely eliminated. The concentration decreases rapidly over the initial 24‐30 hours, although does not reach a low concentration until after ∼2‐3 days. The half‐life survival time of SARS‐CoV‐2 on different materials is shown in Figure 2C, together with the half‐life survival time when the virus is aerosolized.^[^ ^16^ ^]^ SARS‐CoV‐2 can persist on plastic for greater periods than many of the other materials studied, which suggests the virus will also be persistent on polymer composite materials.

The retention of coronavirus and other viruses on plastics have been examined, including SARS‐CoV‐1,^[^ ^16^, ^21^, ^26^, ^27^ ^]^ SARS‐CoV‐2,^[^ ^7^, ^16^, ^19^, ^21^ ^]^ MERS,^[^ ^17^ ^]^ HCoV‐NL63,^[^ ^25^ ^]^ HCoV‐229E,^[^ ^26^ ^]^ and human metapneumovirus.^[^ ^25^ ^]^ The time required for the complete deactivation of different types of coronavirus on plastics is summarized in Figure 2D, under the specified environmental conditions.^[^ ^18^, ^23^, ^28^, ^29^ ^]^ There does not appear to be large differences in the measured survival times of several coronavirus samples on plastic surfaces. There is, however, considerable scatter in the results; SARS‐CoV‐1 has generally been found to be retained on plastic for 3‐4 days, although data exists for viral persistence on plastics anywhere between 1 and 28 days (outlier not shown in Figure 2D). The large scatter is due to the survival time being dependent on multiple factors, including virus initial concentration (viral titer), surface conditions (roughness, charge, and wettability), temperature, and humidity. Most published studies do not specify the type or composition of the polymer used to determine the persistence of viruses, and is generally termed “plastic.” The few studies that do specify the polymer (e.g., PVC, ABS) are not the type used as the matrix phase to composite materials. To the authors’ knowledge, there is no published work into the retention of coronavirus and other virus types on the thermosets (e.g., polyester, phenolic, vinyl ester) or thermoplastics (e.g., PEEK, UHMPE) commonly used in composite materials. Furthermore, it is not known how sensitive the half‐life of viruses is to the chemical composition of the polymer, and whether differences exist between those plastics studied (e.g., PVC) and the polymers commonly used in composite materials.

Nevertheless, the data imply that coronavirus may persist on polymer composite materials for at least 1 day and often for much longer periods under ambient conditions. Unmodified composite surfaces (e.g., not painted or decorated) usually consist of a thin polymer layer (typically less than ∼0.1 mm) or thicker polymer finish such as gel coat (up to 1‐2 mm thick). Therefore, studies into unreinforced plastics could be applied, with care, to potentially understand the retention of viruses on the polymer‐rich surface of composite materials.

## 3 ANTIVIRAL SURFACES AND COATINGS FOR POLYMER COMPOSITES

**FIGURE 3:**
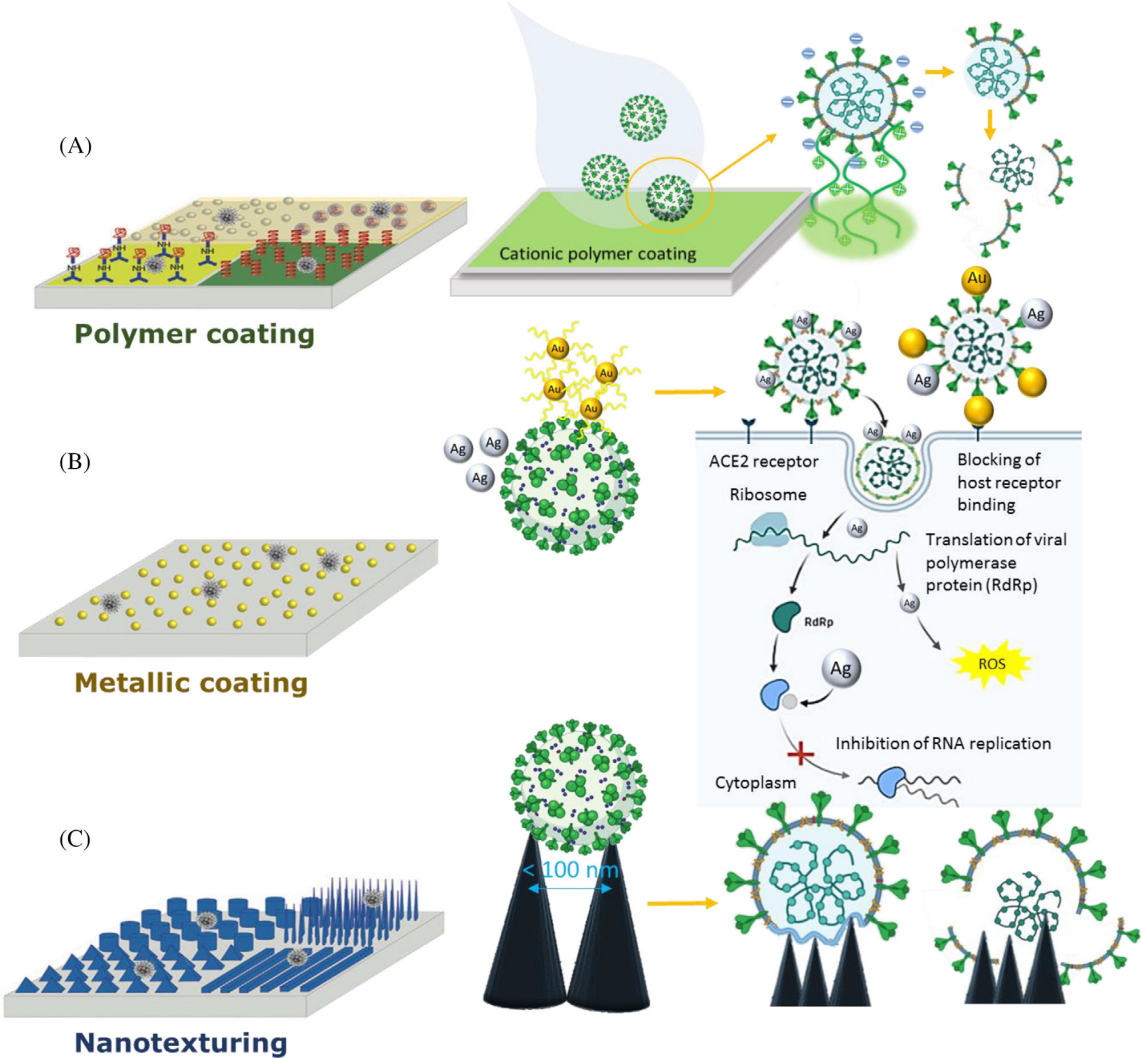
Potential strategies (left) and their corresponding mechanisms (right) for imparting antibacterial and antiviral properties to the surface of polymer composites. A, Polymer coatings that cause cation‐induced disruption of viruses. B, Metallic coatings (including metal nanoparticles) that can block host receptor binding, as well as release ions and reactive oxygen species (ROS) that damage the virus envelope. C, Surface modification by nanotexturing that can deliver mechanical forces to a cell membrane, resulting in cell rupture and death

The methods for creating antiviral polymer composite surfaces that have the ability to reduce the spread of viral pathogens remain largely unexplored. To date, there is no published research into the modification or treatment of the surface of composite materials with a view to reducing the risk of indirect pathogen transmission. However, the scientific literature shows that there are several nanofabrication approaches that could be applied to polymer composites in order to create the antiviral surfaces (Figure 3). These techniques include employment of antiviral compounds (Section 3.1), polymers with intrinsically pathogen‐resistant properties (Section 3.2), metallic surface coatings (Section 3.3), and surface modification of substrata by the process of nanotexturing (Section 3.4). It is worth noting that factors such as virus initial concentration (viral titer), temperature, and humidity are thought to still affect virus retention in the presence of an antiviral surface.

### 3.1 Antiviral compounds

Biocidal doping agents mixed within a polymer surface layer or surface coating offer a potential strategy (albeit untested) to impart biocidal properties to polymer composites. Biocides such as chemical agents (e.g., hydrogen peroxide and formaldehyde), halogen elements (e.g., chlorine and iodine), organic cationic compounds (e.g., quaternized ammonium compounds (QACs) and chitosan), organic non‐cationic compounds (e.g., furanones and triclosan), and other non‐organic compounds (e.g., nitric oxide) could potentially be doped within a polymer to improve its antiviral activity.^[^ ^33^ ^]^ Plasma deposition is the most common technique used to fabricate polymer surface coatings with biocidal doping agents, and can also be used to deposit polymer surface coatings with antiviral nanoparticle doping agents.^[^ ^33^, ^34^ ^]^ Plasma‐deposited polymer surface coatings can also act as a diffusion barrier that controls the release rate of doping agents.^[^ ^34^ ^]^

### 3.2 Antiviral polymers

The polymeric materials that exhibit intrinsic antiviral activity have been identified in numerous studies.^[^ ^35^, ^36^, ^37^, ^38^, ^39^, ^40^, ^41^, ^42^, ^43^ ^]^ The majority of antiviral polymeric materials are applied as surface coatings and do not significantly change the bulk properties of the substrata. When used as coatings, the antiviral polymers are non‐covalently bonded (e.g., painted on) or covalently immobilized onto the substrate. Whether the antiviral polymers are used as the matrix or surface coating, they exert virucidal effects via direct contact with the virus and not through the release of active compounds or agents. Consequently, the antiviral polymeric materials and their active moieties are exposed to the environment and should therefore be resistant to degradation by moisture, temperature and UV exposure as well as being abrasion and erosion resistant. To achieve long‐term uninterrupted antiviral activity, the candidate polymers must be environmentally stable. Thus, chemically attached and high molecular weight virucidal polymers, which are often less toxic to humans and aquatic organisms, are generally preferred.

Several types of polymers exhibit antiviral activity including QACs,^[^ ^36^, ^38^, ^39^, ^43^, ^44^, ^45^ ^]^ quaternary phosphonium or sulfonium derivatives,^[^ ^46^ ^]^ aromatic and heterocyclic derivatives.^[^ ^47^, ^48^, ^49^ ^]^ Notably, the virucidal effects of QACs have been comprehensively studied against a wide‐range of viruses such as *Herpes simplex* virus (HSV), influenza virus, simian 40, varicella zoster, human norovirus (HuNoVs), hepatitis A virus (HAV), hepatitis E (HEV), poliovirus, rotavirus, and human immunodeficiency virus (HIV).^[^ ^46^ ^]^ Quaternized ammonium compounds can be covalently (e.g., chemically bound) or non‐covalently (e.g., painted) bound to different materials, including polymer composites. Moreover, the presence of functional groups often found in polymer composites, such as amine groups in epoxy, promote electrostatic and hydrogen bond interactions with the amino acid residues of reverse transcriptase involved in the viral cycle, leading to antiviral activity.^[^ ^50^ ^]^ The ability to have QACs chemically or physically bound to the composite surface is thought to be critical, as the immobilized quaternary ammonium groups must be accessible to the viral particles. However, this approach has not been studied in the context of polymer composites.

Within the QACs family of antiviral polymeric materials, the *N*‐alkylated derivatives of polyethylenimines (PEIs) have been the most studied.^[^ ^36^, ^37^, ^38^, ^39^, ^41^, ^42^ ^]^ PEIs are organic polymers having repeating units composed of the amine group and ethylene spacers, and they occur as linear or branched polymer chains. Linear PEIs contain secondary amines only while their branched counterparts contain primary, secondary and tertiary amine groups. The presence of amines in PEIs renders them compatible with resin systems, such as epoxy, used as the matrix phase to composite materials. Linear and hyperbranched polyethylenimines have been used as curing and toughening agents for epoxy resins.^[^ ^35^, ^43^ ^]^ The antiviral activity of *N*‐alkylated‐PEIs has been demonstrated against viruses such as influenza virus,^[^ ^37^, ^38^, ^39^, ^41^, ^42^ ^]^ poliovirus,^[^ ^40^ ^]^ rotavirus,^[^ ^40^ ^]^ HIV,^[^ ^36^ ^]^ and HSV.^[^ ^44^ ^]^ All studies reported a relatively strong correlation between the density of quaternary ammonium groups and the antiviral activity. The virucidal potency of *N*‐alkylated‐PEIs is attributed to the quaternized ammonium groups.^[^ ^39^, ^51^ ^]^ As with algae, bacteria and fungi, Hsu^[^ ^39^ ^]^ reported that the viral envelope proteins disintegrate when their negatively charged hydrophobic head groups come into proximity with the positively charged ammonium groups. Other non‐PEI QACs such as *N,N*‐dodecyl,methyl‐polyurethane^[^ ^42^ ^]^ as well as quaternary phosphonium and sulfonium^[^ ^46^ ^]^ and poly(4‐vinylpyridine)^[^ ^52^ ^]^ derivatives also inactivate enveloped viruses (e.g., influenza and poliovirus) based on the antiviral mechanism involving the cation‐induced viral disruption of the membrane (Figure 3A).^[^ ^52^ ^]^

Heterocyclic and aromatic polymeric materials exhibiting antiviral properties include phenolic^[^ ^49^ ^]^ and benzoic acid derivatives.^[^ ^48^ ^]^ The humic acid‐like *p*‐diphenolic compounds derivatives hydroquinone, 2,5‐dihydroxytoluene, and 2,5‐dihydroxybenzoquinone have been used to inhibit replication of the herpes virus.^[^ ^48^ ^]^ The *p‐*diphenolic polymers interact with positively charged domains of viral envelope glycoprotein causing cell death through disintegration of the cell membrane and the release of intracellular material. Moreover, *p‐*diphenolic are compatible with polymers with functional hydrophilic end groups (such as epoxy resins) as evident from novolac epoxy resin formulations (a combination of phenol, methanal (formaldehyde) and epoxy moieties). Benzophenone ester and benzophenone amide are other examples of heterocyclic compounds compatible with engineering polymers and exhibiting antiviral activity against influenza.^[^ ^47^ ^]^ Benzophenone is already a common additive to plastic packaging films as a UV blocker to prevent photo‐degradation and does not pose toxicity issues against humans.^[^ ^46^ ^]^

Polysaccharides are another class of polymeric materials exhibiting antiviral activity.^[^ ^51^, ^53^ ^]^ Several studies have demonstrated the antiviral ability of chitosan and its derivatives against human enteric viruses (e.g., HuNoV and HAV),^[^ ^54^ ^]^ influenza viruses,^[^ ^55^, ^56^ ^]^ HSV and HIV.^[^ ^57^ ^]^ Chitosan, chitin sulfated derivatives or its conjugated complexes inhibit viral‐host cell binding events and replication.^[^ ^51^, ^55^, ^56^, ^57^ ^]^ While polysaccharides have demonstrable viricidal effects on many virus types, they do not have the adequate mechanical properties to be independently used as matrices in structural composite materials, though could be used as a non‐structural coating. Thus, antiviral polysaccharides can be integrated as fillers in thermoplastic and thermoset resins or applied as surface coating on polymer composites. Surface coating is the preferred polysaccharide integration method as it does not adversely impact the mechanical properties of the composite. The layer‐by‐layer (LbL) coating technique, which involves repeated immersion‐drying of the composite into a solution containing chitosan or its derivatives, is a promising method to achieve uniformity in virucidal efficacy on surfaces.^[^ ^58^ ^]^

### 3.3 Metallic surface coatings

Metal‐based surface coatings represent a promising strategy for imparting biocidal properties to polymer composite materials. Metals and their oxides (including gold (Au), silver (Ag), aluminium (Al), copper (Cu), iron oxide (Fe_2_O), zinc oxide (ZnO), magnesium oxide (MgO) and titanium dioxide (TiO_2_)), in either bulk form or nanoparticle form, have been reported to exhibit antiviral properties.^[^ ^59^, ^60^, ^61^, ^62^, ^63^, ^64^, ^65^, ^66^, ^67^ ^]^ In particular, viruses in contact with bulk Ag, Al, Cu and Zn surfaces have demonstrated reduced infectivity in comparison to those viruses retained on plastic surfaces in the absence of these metals. For example, SARS‐CoV‐1^[^ ^16^ ^]^ and CoV‐229E^[^ ^18^ ^]^ have exhibited short persistence times (usually less than several hours under ambient conditions) on Cu surfaces. Sizun et al.^[^ ^15^ ^]^ reported that HCoV‐OC43 and HCoV‐229E were viable on Al for less than 2 and 6 hours, respectively (considerably shorter than their half‐life on a plastic surface, Figure 2). Similarly, HCoV‐229E was found to persist on Zn for only 2 hours.^[^ ^18^ ^]^ When in contact with Cu and brass, viruses such as HuCoV‐229E are inactivated by Cu ions and reactive oxygen species that have the capacity to damage the virus envelope (Figure 3B).^[^ ^8^, ^18^, ^68^, ^69^, ^70^ ^]^ In bacteria, Cu induces an oxidation‐reaction stress that damages the cell membrane, alters the conformational structure of proteins, and degrades the cellular DNA and/or RNA.^[^ ^65^, ^71^, ^72^, ^73^ ^]^ Similar mechanisms for inactivating viruses may exist for other heavy metal ions.^[^ ^68^, ^70^ ^]^ Recently van Doremalen et al.^[^ ^16^ ^]^ and Suman et al.^[^ ^7^ ^]^ demonstrated that Cu can inactivate SARS‐CoV‐2 in less than ∼1 hour. Nevertheless, not all metals have been found to be as effective in the rapid inactivation of viruses; for example van Doremalen et al.^[^ ^16^ ^]^ and Chin et al.^[^ ^28^ ^]^ reported, respectively, that SARS‐CoV‐2 can persist on stainless steel surfaces (which is bioinert) for 3 and 4 days. MERS‐CoV can remain viable on stainless steel for about 1 day under relatively low humidity conditions.^[^ ^17^ ^]^

The extensive research that has been performed into the antiviral properties of metals indicates that they could impart similar properties to polymer composites when used as a thin metal coating or metal‐enriched surface layer. This approach has already been demonstrated for anti‐bacterial properties, with several studies coating polymers with a thin layer of copper to impart bacterial resistance.^[^ ^71^, ^74^, ^75^ ^]^ It is, therefore, feasible that thin metal and metal oxide coatings could be used to provide polymer composite materials with the ability to resist viruses, although this has not yet been evaluated. Thin metal coatings can be applied to polymer composites using techniques such as cold spray,^[^ ^76^, ^77^, ^78^ ^]^ pulsed gas dynamic spray,^[^ ^79^ ^]^ magnetron sputtering,^[^ ^80^ ^]^ detonation gun spraying^[^ ^81^ ^]^ and 3D printing.^[^ ^82^ ^]^ A continuous metallic film should be sufficient to impart anti‐viral properties to polymer composite materials. The required thickness of the metallic coating will likely depend on other physical requirements of the end application. The use of metal or metal oxide coatings on polymer composites can, however, cause inherent problems, possibly including low bond strength and residual strains within the coating leading to cracking and spalling, high cost (particularly when using expensive heavy metals), durability (e.g., abrasion resistance) and galvanic corrosion. Published research regarding the use of metal coatings to reduce the retention of viruses on polymers and polymer composites is lacking.

In addition to continuous metal films, metal nanoparticles have the potential to impart biocidal properties to polymer composite materials. The potential use of nanoparticles as agents to immobilize and inactivate SARS‐CoV‐2 has been recently reported.^[^ ^59^, ^83^ ^]^ An analysis of published research reveals that spherical nanoparticles are likely to demonstrate greater (∼123%) antiviral activity against coronaviruses (MERS‐CoV and SARS‐CoV‐2) compared with particles possessing other nanomorphologies (e.g., rods and spikes).^[^ ^59^ ^]^ The exact mechanism of biocidal activity remains poorly understood^[^ ^60^ ^]^ since the variability in parameters such as concentration, dimensions, physical and chemical properties, as used in different studies, have not produced consistent results. This is partly due to the multifaceted nature of the biocidal activity, which makes it difficult to decouple the individual mechanisms taking place.

Ag, Cu, gallium (Ga) and Au nanoparticles all exhibit strong biocidal activity.^[^ ^59^, ^60^, ^61^, ^62^, ^63^, ^64^, ^65^, ^66^, ^67^ ^]^ Ag nanoparticles (AgNPs) are the most widely studied for their antiviral activity.^[^ ^60^, ^84^, ^85^, ^86^, ^87^ ^]^ AgNPs adhere onto the viral lipid envelope (specifically, the viral envelope glycoproteins) and then block receptor‐mediated interactions (CD4‐dependent virion binding events) between the virus and host cell.^[^ ^59^, ^84^, ^85^ ^]^ Graphene‐Ag nanocomposites have also been reported to inhibit the adhesion and replication of viruses.^[^ ^88^ ^]^ (Graphene alone is another potential antiviral nanomaterial due to its large surface area, high carrier mobility and biocompatibility^[^ ^59^ ^]^). Borkow and Gabbay^[^ ^89^ ^]^ demonstrated the potential of doping metal particles into polymers to impart biocidal properties.^[^ ^89^ ^]^ They blended a low concentration of Cu oxide particles (70% Cu_2_O and 30% CuO) into latex and polyester fibers, and demonstrated that these were effective in reducing the infectivity of the HIV‐1 virus. Similarly, functionalized rod‐shaped AuNPs were shown to actively inhibit the replication of the measles virus (MeV),^[^ ^90^ ^]^ MERS‐CoV^[^ ^91^ ^]^ and SARS‐CoV.^[^ ^92^ ^]^ The effective size of the AgNPs plays an important role in their antiviral efficacy;^[^ ^84^, ^86^, ^87^ ^]^ AgNPs that are 1‐10 nm in size are thought to be more likely to bind to the surface of an enveloped virus; thus, inhibiting their ability to enter host cells.^[^ ^86^ ^]^

Metallic, carbon and silica nanoparticles (among others) are commonly blended into the polymer matrix of composite materials to improve multiple properties including mechanical strength,^[^ ^93^, ^94^, ^95^, ^96^ ^]^ fracture toughness,^[^ ^97^, ^98^ ^]^ electrical conductivity,^[^ ^93^, ^97^, ^99^ ^]^ thermal diffusivity^[^ ^99^ ^]^ et. The maximum concentration of nanoparticles used to do this is typically less than 2‐5 vol% due to problems of very high resin viscosity; however, this is expected to be insufficient to impart these composite materials with improved antiviral activity. A high concentration of nanoparticles may be required within the near‐surface layer of composite materials, and this could be achieved by applying cold spray or other deposition techniques prior to the resin cure stage. The efficacy of a metal nanoparticle‐enriched surface layer to impart biocide properties to polymer composites has, however, not been investigated.

### 3.4 Antiviral surface structure modifications by nanotexturing

Pioneering work by the authors has proved that the nanostructured surfaces of insect wings can exert mechanical forces upon a cell membrane, resulting in cell rupture and death^[^ ^100^, ^101^ ^]^ (Figure 3C). Drawing inspiration from nature, the topographical modification of synthetic materials to impart antimicrobial capabilities can be achieved by nanostructuring the surface of substrata. Nanotextured surfaces made of an array of nanoprotrusions with 100‐1000 nm separation and comparable height can kill bacteria upon physical contact (in air, or in water on hydrophobic or hydrophilic surfaces). Adhesion of the bacterial membrane to the nanostructure array has been shown to induce cell rupture as the membrane is stretched beyond its elastic limit. A broad variety of nanopatterns produced on different materials can induce bacterial cell death according to the mechano‐bactericidal mechanism described above. For example, the surface topographical modification of materials such as ceramics, carbon, polymer, metal and metal oxides to impart nanopatterns composed of an array of nanopillars, nanocolumns, nanowires, nanospinules, nanospikes, and nanocones have all demonstrated the nanostructure‐induced rupture of attaching bacterial cells.^[^ ^102^, ^103^, ^104^ ^]^ The design and manufacture of these mechano‐bactericidal surface topographies has been explored, for the most part, as an alternative method of preventing the bacterial contamination of medical devices and orthopaedic implants in response to the rising number of infections caused by antibiotic‐resistant bacterial strains.^[^ ^102^ ^]^ For example, highly efficient mechano‐bactericidal surface topographies are created in the fabrication of infection‐resistant biomedical titanium implants.^[^ ^100^, ^103^ ^]^ The principle of killing bacteria by physical rupturing using nanostructured surfaces could be readily transferred to the mechanical inactivation of viruses by rescaling of the surface topographic dimensions to be applicable to that of viruses. Moreover, nanostructured aluminum Al 6063 alloy surfaces were recently demonstrated to inactivate SARS‐CoV‐2 after 6 hour of exposure.^[^ ^105^ ^]^ Thus, surface micro‐nanostructuring may offer a safe alternative to imparting antiviral and antibacterial properties to the surface of polymer composite materials, although this has yet to be demonstrated.

Some enveloped viruses (including SARS‐CoV‐2 and influenza) are approximately 100 nm in diameter, and hence inactivation of a 100 nm virus could be conceptually considered as a scaling problem to the inactivation of bacteria (1000 nm in diameter) on nanotextured surfaces, demonstrated earlier on natural and engineered surfaces, including metallic and polymeric materials.^[^ ^102^, ^106^ ^]^ The generic principle of bactericidal action based on mechanical action of the nano‐textured surface is grounded in the tensile stresses exerted on the bacterial membrane.^[^ ^102^, ^107^ ^]^ The direct contact point stress, tensile stress between neighboring contact points, and the flexural stress of the bended pillar exerted onto the contact point with bacteria, are all different modalities of action that define the mechano‐bactericidal function of nanostructured surfaces.^[^ ^102^, ^107^ ^]^ On the nanoscale, forces change their dominance and as the size of an object is decreased towards molecular dimensions, and therefore van der Waals and other molecular forces become increasingly dominant.^[^ ^108^ ^]^ The practical work of adhesion against a nanostructured surface may be sufficient to trap a virus and exceed its envelope rupture stress, which is expected to scale as for bacterial membranes: *L^2^ T^–2^*, where *L* and *T* are the length and thickness, respectively. Stress is defined by *σ = E ΔL/L*, hence for the same material (Young modulus *E*(virus) = *E*(bacteria)) the same strain (relative elongation) will create the same stress which has been found to be lethal for bacteria.^[^ ^109^ ^]^

Our initial studies of the antiviral efficacy of nanostructured surfaces show that surfaces possessing sharp, dense nanopillars made of black silicon have the capacity to rupture the influenza virus.^[^ ^107^ ^]^ Another study has reported that Al surfaces with a nanoscale roughness ranging from ∼70 to 1000 nm exhibited antiviral activity against both enveloped (respiratory syncytial virus) and non‐enveloped (rhinovirus) viruses.^[^ ^62^ ^]^ Nanostructured surfaces coated with TiO_2_ are also promising biocidal surfaces that exhibit strong oxidizing (electron removal) properties (with UV light activation) that can be used to kill attaching viral particles.^[^ ^110^ ^]^

The shape of the wetted perimeter of respiratory droplets will be significantly different across various nanostructured surfaces according to their respective hydrophobic/hydrophilic characteristics. This is likely to affect the drying rates of the respiratory droplets, which will lead to altering the (meta)stability of extending and reshaping the droplet interface, creating gradients in Laplace pressure that could also influence evaporative drying rates and viral viability.

The fabrication of effective biocidal nanopatterns on the surface of polymer composite materials is a challenging task. Current popular nanofabrication methods for the manufacture of bactericidal nano‐topographies include both top‐down and bottom‐up nanofabrication techniques such as plasma techniques, reactive ion etching, hydrothermal treatment, anodizing, chemical etching, electrodeposition, and chemical vapor deposition.^[^ ^45^ ^]^ Plasma techniques are the most common method for creating nanostructured surfaces in polymers and polymer composites.^[^ ^34^, ^111^, ^112^, ^113^, ^114^ ^]^ Plasma surface treatments can be used under ambient temperature conditions, are highly reproducible, do not significantly change the bulk material properties, and produce more consistent surface finishes compared with chemical and mechanical processes.^[^ ^112^ ^]^ Nanostructuring the substrata surface by plasma etching allows for the control of the surface‐to‐volume ratio, surface energy, aspect ratio of surface geometry, light absorbance, surface functionalization, and size effects. Plasma treatments have been used to improve adhesion between fibers and matrices to enhance the mechanical properties of polymer composites,^[^ ^111^ ^]^ and have also been used to create a hydrophobic surface with reduced surface wettability to improve biological functioning of polymer composite bone implants.^[^ ^114^ ^]^ In addition to plasma treatments, nanoimprint lithography (NIL) is a viable method for the creation of nanostructured topologies on polymer surfaces.^[^ ^104^, ^107^ ^]^ In this method, negative molds/templates composed of ceramic or metal are used to imprint a pattern onto a polymer resin that is then cured using heat or UV.^[^ ^107^ ^]^ It is feasible that NIL could be adapted to create pathogen‐resistant topographies on polymer composite surfaces. NIL has already been used to modify the surface of polymer composite materials to improve their bond strength as joints.^[^ ^115^, ^116^ ^]^ For example, Matsuzaki and Suzuki^[^ ^116^ ^]^ used NIL to create a pyramidal microstructure on the surface of a polymer composite butt joint prior to adhesive bonding, improving the strength by 67% compared to the joint without nanoimprints.

## 4 CONCLUDING REMARKS

The growing use of polymer composite materials in “high‐touch” products combined with the global COVID‐19 pandemic have highlighted the emerging need for antiviral surfaces. Many types of viruses, including coronavirus, can persist and retain their infectivity for several days on plastics under ambient conditions. These relatively long retention times increase the risk of indirect transmission of the virus between humans via plastic surfaces. The surface of composite products is usually polymer‐rich, and therefore it is likely that many viruses, including SARS‐CoV‐2, could survive for a prolonged period on such surfaces and therefore, possibly aid the indirect transmission of viruses. The persistence of viruses on commonly‐encountered thermoset and thermoplastic composites has not been investigated thus far. There is a need to measure the persistence of different virus types on commonly encountered composite materials such as carbon‐epoxy and glass‐polyester laminates. Such research is critical for the greater understanding of the risks associated with the indirect transmission of viruses via these composite surfaces.

A large body of published research exists on strategies designed to shorten the half‐life of viruses and bacteria on polymer surfaces, and these strategies have the potential to be applied to polymer composites. The addition of biocidal polymer matrices, metal and metal oxide coatings, graphene or metallic nanoparticle‐enriched polymers, and nanostructured textures (independently or in combination) to polymer composite materials are approaches that have the potential to produce antiviral materials. Published research demonstrating the efficacy of these strategies when applied to polymer composites is, however, lacking, and is a topic worthy of investigation. In addition to biocidal efficacy, a holistic assessment of these strategies when applied to polymer composite substrata is needed, with consideration being given to other important factors such as cost, ease of fabrication, surface durability and aesthetics. The SARS‐CoV‐2 global pandemic has delivered a strong message to the polymer composites community that opportunities exist for the creation of next‐generation materials possessing virus‐resistant surfaces.

## CONFLICT OF INTEREST

The authors declare no conflict of interest.
